# Fracture Analysis of Concrete Structures: Prediction Based on Boundary Effect Model

**DOI:** 10.3390/ma18081877

**Published:** 2025-04-20

**Authors:** Gang Han, Xiangyu Han, Yi Ji, Xiaozhi Hu

**Affiliations:** 1 Department of Mechanical Engineering, University of Western Australia, Perth, WA 6009, Australia; gang.han@research.uwa.edu.au; 2 School of Civil Engineering and Architecture, Southwest University of Science and Technology, Mianyang 621010, China; xiangyuhan03@163.com; 3 School of Manufacturing Science and Engineering, Southwest University of Science and Technology, Mianyang 621010, China; jiyi@swust.edu.cn

**Keywords:** predictive fracture model, boundary effect model (*BEM*), fracture process zone (*FPZ*), aggregate size, size effect

## Abstract

A simple design model, able to link test results of small concrete samples to failures of large structures, is desirable for fracture analysis of concrete structures, particularly if the model has no special requirements on small samples, e.g., size, notched or un-notched. The linear Boundary Effect Model (BEM, which has evolved over the past 20 years, is able to provide the link between small samples and large structures with fairly reliable predictions and a built-in function of statistical analysis. BEM enables researchers and engineers to model the quasi-brittle fracture behavior of concrete and the associated size effects by focusing on the fracture process zone (FPZ) at the notch tip or at the specimen boundary (for an un-notched case). FPZ and quasi-brittle fracture of concrete are directly influenced by the average aggregate size ($d_{av}$), but few models mathematically show such critical aggregate influence, except BEM. The aggregate size used in BEM can be accurately estimated separately before fracture experiments. A comprehensive dataset of concrete fracture results from the existing literature, along with a new experimental dataset from three-point bending (3-P-B) tests, involving 138 specimens with varying notch depths (un-notched, 1 mm shallow-notched, and 6 mm deep-notched) was analyzed. The specimens, which present inconsistent dimensions (160 mm span, approximately 40 mm thickness, and 38 mm width/height), were used to estimate FPZ at peak fracture loads and investigate their interactions with structural boundaries. Statistical analyses were integrated into BEM, allowing the model to account for the experimental scatter, thus improving its reliability as a predictive tool for maximum fracture loads of concrete structures. This study confirmed again that the linear BEM is easy to use and provides fairly accurate predictions across concrete specimens and structures of various sizes.

## 1. Introduction

Being the most widely used engineering material, concrete and concrete structures are fundamental to diverse structural applications, where their reliability directly impacts the overall cost and safety. Different from homogeneous materials, which exhibit consistent and predictable responses to applied forces, concrete is a large particle composite characterized by a mixture of aggregates, sands, cement paste, and interfacial transition zones so that concrete fracture is quasi-brittle [1]. This composite structure with heterogeneous characteristics typically displays complex fracture behavior, resulting in nonuniform stress distributions and multiple micro-fractures. Therefore, it is challenging to predict the critical fracture or safety load of a large concrete structure based on experimental observations from small test samples in laboratories.

Without a doubt, conventional fracture mechanics like Linear Elastic Fracture Mechanics (LEFM), as expressed by the standards [2,3], are inherently inappropriate for concrete analysis and predicting the behavior of concrete structures. This is because LEFM is designed for linear-elastic and homogeneous materials without pre-existing micro-damages or cracks. Hence, it is critical to develop and adopt more advanced analytical approaches that can accurately capture the unique properties and fracture behavior of concrete, ultimately providing more reliable predictive models for practical applications. Furthermore, a simple design model is expected to predict the failures of large concrete structures —whether or not visible cracks are present—based on experimental data obtained from small notched three-point bending (3-P-B) samples made of the same concrete mix. Practically, these small specimens are cost-effective to prepare and easy to handle. Moreover, variable notch sizes can be easily designed on small samples, with a particular feature that notch determines the fracture location for the stress analysis and modeling of the crack-tip fracture process zone (FPZ).

The well-known Dugdale–Barenblatt model [4,5] was initially developed for metals containing elastic plastic fracture features. Similarly, the initiation and development of FPZ during quasi-stable crack growth was then assessed using the fictitious crack model in 1976 [6] and further developed by employing $K_{R}$-curve model in [7,8]. Recently, the size effect induced by the fracture process zone (FPZ) on various fracture behaviors, ranging from small specimens to large structures, has garnered significant attention in the concrete fracture community following foundational studies issued in 1983 and 1984 [9,10].

Since then, the Size Effect Law (SEL) has become a widely recognized framework for modeling quasi-brittle fracture of concrete. It comprises three distinct equations tailored for unnotched, shallow-notched and deep-notched specimens, incorporating over ten fitting parameters [11,12,13]. This complexity can restrict its applicability in broader studies. Nonetheless, two independent research teams have conducted extensive tests that confirm the SEL’s effectiveness in characterizing the quasi-brittle fracture behavior of concrete and interpreting the associated size effects [14,15]. Given these findings, the development of simplified equations with fewer parameters represents a promising path for providing a more practical approach for analyzing size effects in concrete fracture.

In pursuit of reducing the complexity inherent in the SEL, a significant breakthrough was reported in the form of the Boundary Effect Model (BEM), which can serve as an alternative to the SEL, as demonstrated by Chen and Hu [16]. The BEM is represented by a single linear curve that intersects the origin (0,0), with the slope defined by the intrinsic tensile strength $f_{t}$. Despite its simplicity, the BEM effectively models the entire range of quasi-brittle fracture behavior for unnotched, shallow-notched, and deep-notched specimens of varying sizes.

This innovative approach allows for the replacement of three different SEL equations with one linear BEM applicable to both geometrically similar specimens and those of uniform size, regardless of whether they exhibit interfacial cracks or notches. Consequently, future size effect experiments and analyses are anticipated to become significantly more straightforward, as researchers will no longer be constrained by the need for geometrically analogous specimens across a wide range of sizes, a requirement that SEL typically imposes.

To further investigate and validate the new linear Boundary Effect Model (BEM), this study conducted a comprehensive series of 138 tests. The primary objective was to demonstrate that the study of the concrete size effect can be significantly simplified, as discussed previously, both experimentally and analytically, through the use of the BEM for small-scale samples. The linear BEM can subsequently be applied to predict the fracture (or safe) loads of larger concrete beams. This study included three new sets of three-point bending (3-P-B) tests featuring unnotched, shallow-notched, and deep-notched specimens, all of similar geometry ($160\times 40\times 38\;{mm}^{3}$). The results from these tests were all analyzed using the newly developed linear BEM.

## 2. Linear Formulation for 3-P-B Geometry: Insights into Fracture Mechanics and Predictive Modeling

### 2.1. The Linear Boundary Effect Model (BEM): Core Concepts and Formulation

The new linear BEM [16,17,18] recently proposed and experimentally verified, is to correlate the maximum fracture load $P_{max}$ of a 3-P-B specimen with the tensile strength $f_{t}$ (the criterion for FPZ formation) and an equivalent area $A_{e}$:

(1) $$P_{max}=f_{t}\times A_{e},$$

where $A_{e}$ is the equivalent area, which is independent of specimen geometric dimensions. It is completely determined using the specimen measurements and the average aggregate size $d_{av}$. In particular, for the 3-P-B geometry, span S, thickness B, and notch width/height ratio ($\frac{a_{0}}{W}$) are integrated for the equivalent area $A_{e}$ estimation:

(2) $$A_{e}=\frac{W^{2}\times \left(1-\frac{a_{0}}{W}\right)\times \left(1-\frac{a_{0}}{W}+\frac{2{FPZ}_{L}}{W}\right)}{1.5\left(\frac{S}{B}\right)\sqrt{1+\frac{a_{e}}{{FPZ}_{W}}}}=\frac{W^{2}\times \left(1-\frac{a_{0}}{W}\right)\times \left(1-\frac{a_{0}}{W}+\frac{3d_{av}}{W}\right)}{1.5\left(\frac{S}{B}\right)\sqrt{1+\frac{a_{e}}{3d_{av}}}}$$

where the equivalent size $a_{e}\;$ is determined by:

(3) $$a_{e}={\left[\frac{{\left(1-\frac{a_{0}}{W}\right)}^{2}\times Y\left(\frac{a_{0}}{W}\right)}{1.12}\right]}^{2}\times a_{0}\;$$

A given notch length $a_{0}$ has different effects on fracture depending on whether the specimen is large or limited in size. The equivalent size $a_{e}$ in Equation (3) specifies the difference. Classic LEFM considers the influence of the notch-to-height ratio $\frac{a_{0}}{W}$ through the geometry factor $Y(\frac{a_{0}}{W})$, as demonstrated in Equation (4), which is utilized in this research for small-size specimens. Specifically, for a large geometry plate where $\frac{a_{0}}{W}\to 0$, the geometry factor Y=1.12 [19], so the equivalent size $a_{e}=a_{0}$ and the large plate solution is recovered.

(4) $$Y\left(\frac{a_{0}}{W}\right)=\frac{1-2.5\left(\frac{a_{0}}{W}\right)+4.49{\left(\frac{a_{0}}{W}\right)}^{2}-3.98{\left(\frac{a_{0}}{W}\right)}^{3}+1.33{\left(\frac{a_{0}}{W}\right)}^{4}}{{\left(1-\frac{a_{0}}{W}\right)}^{\frac{3}{2}}}$$

Notably, in Equation (2), FPZ at the notch tip is considered to correlate to the average aggregate size $d_{av}$, with a formulation of ${FPZ}_{W}=2{FPZ}_{L}=3d_{av}$. Moreover, the FPZ length and width ratios ($\frac{{FPZ}_{L}}{W}$, $\frac{a_{e}}{{FPZ}_{W}}$) in comparison to the notch width/specimen height ratio and notch $a_{0}$ (or $a_{e}$) were also considered. The linear function in Equation (1) is based on the original asymptotic analysis for a large plate with a short edge crack ($\frac{a_{0}}{W}\ll 1$) or “boundary effect model” (BEM) [19].

It is worth mentioning that all parameters associated with the equivalent area $A_{e}$ are geometry variables, which are then interpreted into geometry constants with measurable sizes. However, the average aggregate size $d_{av}$ of concrete is a material variable that should be known and specified.

Moreover, one critical feature of the core linear theory of BEM is to account for the impact of aggregate size on the fracture strength $f_{t}$ and fracture toughness $K_{IC}$ of the highly heterogeneous composite structures. Reasonably, $d_{av}$ could be simply sourced from the vendor datasheet of specific concrete mix products and resultant aggregate structures of concrete specimens. Instead, it can also be feasibly calculated through BEM if relevant information is absent.

The determination of $d_{av}$ is essential for distinguishing concrete properties, as it can vary even when the maximum aggregate size $d_{max}$ remains constant across different concrete mixes. Once the average aggregate size $d_{av}$ is established before fracture testing (or determined using BEM after testing), the only additional constraint required in the linear function for the three-point bending (3-P-B) geometry is the tensile strength $f_{t}$, which serves as the local criterion for FPZ formation at the notch or crack tip.

Alternatively speaking, if the tensile strength $f_{t}$ for a specific concrete mix is identified, the maximum fracture load $P_{max}$ for large 3-P-B constructions can be calculated (predicted) directly using this simple linear function represented in Equation (1) for any crack with notch size $a_{0}\geq \;0$. This implies that the width W in Equation (1) could be small for lab-available investigating samples or larger when applied to engineering constructions.

### 2.2. “Hall-Petch” Relation for Brittle Solids to Link d_av_ with f_t_ & K_IC_

In the classic Hall–Petch relation [20,21] for metals, the yield strength $\sigma_{Y}$ and average grain size $d_{G}$ are linked together as follows:

(5) $$\sigma_{Y}=\sigma_{0}+k\times \frac{1}{\sqrt{d_{G}}}\;$$

This relation can only conduct a case-by-case study for a specific metal, i.e., curve fitting is required to determine the two fitting parameters, $\sigma_{0}$ and k, for the given metal.

BEM has recently established a parallel “Hall-Petch” relationship for brittle heterogeneous solids such as concrete, rock and ceramics [18,22,23], which helps derive the linear BEM in Equation (1):

(6) $$K_{IC}=2f_{t}\sqrt{3d_{av}}\;or\;f_{t}=0+\frac{K_{IC}}{2\sqrt{3}}\times \frac{1}{\sqrt{d_{av}}}$$

By comparing Equations (5) and (6), it becomes obvious that because $\sigma_{0}=0$, the new “Hall-Petch” relationship for brittle materials contains only one parameter, and the previously unidentified parameter k is now characterized by the fracture toughness $K_{IC}$. The correlation between $f_{t}$ and $K_{IC}$ in Equation (6) elucidates why Equation (1) applies to both notched and unnotched specimens.

Proposed in the early 1950s and garnering over 9000 citations, the classic Hall-Petch relation is limited to grain sizes ranging from 20 nm to 200 µm. In contrast, the new “Hall-Petch” equation for brittle substances and composites is valid for microstructures ranging from atomic scales (less than 1 nm) up to 200 mm for large engineering constructions, such as concrete dams [18]. Equation (6) remains applicable across a wide variety of brittle solids, including single-crystal silicon, fine and coarse ceramics, rock, concrete, bone, and fiber-reinforced composites [18,23,24,25]. The introduction of this new “Hall-Petch” relation has resulted in the simplification of three SELs into a single linear formula, as described in Equation (1).

Equation (2) demonstrates how Equation (1) functions both the width and length of the FPZ and its connection to the average aggregate size $d_{av}$. For larger sizes W, $\frac{{FPZ}_{L}}{W}$ could be neglected, allowing us to focus solely on the $\frac{a_{0}}{{FPZ}_{W}}(a_{e}=a_{0})$. Thus, only the crack blunting effect of ${FPZ}_{W}$ must be studied for large constructions.

If $a_{e}=a_{0}=0$, Equation (1) simplifies to the following:

(7) $$P_{max}=f_{t}\times \frac{W^{2}\times \left(1+\frac{{3d}_{av}}{W}\right)}{1.5\left(\frac{S}{B}\right)}\;$$

Notably, if $\frac{d_{av}}{W}=0$, this leads to the classic result from stress analysis:

(8) $$P_{max}=\sigma_{N}\times \frac{W^{2}}{1.5\left(\frac{S}{B}\right)}$$

To achieve a 10% error tolerance using conventional stress analysis for homogeneous materials, it should be guaranteed that $\frac{{3d}_{av}}{W}<0.1$ or $W>30d_{av}$. Under these restrictions, heterogeneous concrete may be approximated as a “homogeneous material.” Recently, Equation (7) has been validated for ordinary concrete specimens without notches, further supporting the ASTM standard for three-point bending tests on laminated carbon fiber composites [26].

### 2.3. Linear Function with Statistical Reliability

Measurements of tensile strength $f_{t}$ obtained from direct tensile exams can be significantly affected by pre-existing micro-defects [27]. Consequently, the presence of weak sections—such as micro-pores, weak interfaces, and suboptimal aggregate or sand structures—at specific locations can lead to a higher intrinsic $f_{t}$ (necessary for FPZ formation) compared to the tensile strength $f_{t-tensile}$ measured through direct tensile tests. However, Equation (1) offers an alternate approach for estimating the intrinsic $f_{t}$ from small three-point bending (3-P-B) samples, where the $f_{t}$ estimation is derived directly from the maximum fracture load $P_{max}$.

The heterogeneous aggregate structures at the notch tip may vary from specimen to specimen, and scatters in fracture loads are inevitable. The normal distribution with a reliability band is adopted by utilizing the average $\mu \;(=f_{t})$ and standard deviation σ. Equation (1) is able to then be altered as follows:

(9) $$P_{max}=\left(\mu \pm 2\sigma \right)\times A_{e}$$

**Figure 1:**
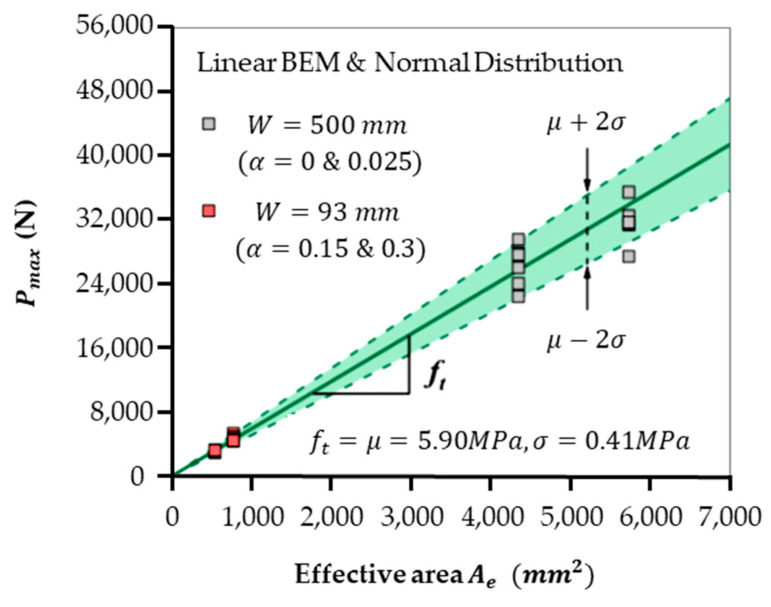
Predictions for large un-notched and shallow-notched concrete beams ($\frac{a_{0}}{W}=0$ and 0.025) of size W=500 mm are compared with similar results using small deep-notched samples ($\frac{a_{0}}{W}=0.15$ and 0.3) that have a size of W=93 mm.

To the best of our knowledge, BEM is the only fracture mechanics model that has the built-in function of statistical analysis, as shown in Figure 1. For instance, the well-received SEL [11,12] can only be used to generate a fitted curve through scattered data without any reliability band.

It is important to emphasize that there is no specific requirement for the initial notch $a_{0}$ in this linear BEM formulation. The initial notch can be zero in the case of un-notched specimens or greater than zero for shallow- and deep-notched cases. This means that the three SELs for un-notched, shallow-notched, and deep-notched specimens have effectively been consolidated into a single linear equation represented in Equation (1). Additionally, the stringent requirement for geometrically similar specimens, which is typically necessary for size effect tests, is no longer applicable. The statistical reliability range indicated in Equation (9) extends beyond the considerations of the SELs, which themselves involve more than ten fitting parameters.

Therefore, if the average tensile strength $f_{t}$ is obtained through small specimens in the laboratory, the allowable maximum load on a large engineering geometry can be predicted within a reliable band of $\left(f_{t}-2\sigma \right)\times A_{e}$ and $\left(f_{t}+2\sigma \right)\times A_{e}$. This reliable prediction does not require curve fitting, but a 95% reliability can also be guaranteed.

### 2.4. Comprehensive Results from the Literature in Statistical Reliability Analysis

In the recent literature [14], a set of 3-P-B tests utilized specimens of diverse sizes, specifically with widths (W) of 40 mm, 93 mm, 215 mm and 500 mm. To examine the influence of initial notch characteristics, the tests covered a wide range of $\frac{a_{0}}{W}$ ratios, spanning from un-notched specimens ($\frac{a_{0}}{W}=0$) to shallow-notched ($\frac{a_{0}}{W}=0.025\;\&\;0.075$) and deep-notched specimens ($\frac{a_{0}}{W}=0.15\;\&\;0.3$). Based on these parameters, five sets of geometrically similar specimens were prepared, each maintaining consistent $\frac{a_{0}}{W}$ ratios while varying specimen sizes. Afterward, in order to validate the effectiveness of BEM and compare the analytical results with SEL outputs, the specimens were also regrouped into four sets to maintain a constant specimen size in the literature [16]. Hence, a total of nine sets of comprehensive data for concrete fracture can be utilized to examine the applicability of this fracture model.

Notably, these specimens demonstrated flexible grouping methodologies beyond size and geometry alignment. For example, a mixed grouping strategy combining small deep-notched specimens ($\frac{a_{0}}{W}=0.15\;\&\;0.3$, W=93 mm) with large un-notched and shallow-notched specimens ($\frac{a_{0}}{W}=0\;\&\;0.025$, W=500 mm). This diverse experimental approach aimed to explore whether small specimen behavior could predict fracture properties of larger, non-geometrically similar concrete structures, as demonstrated in Figure 1. The agreements between small samples of 93 mm and large specimens (or “structures”) of 500 mm, as shown in Figure 1, are significant, as this simple linear relation is ideal for practical engineers and PhDs working in the field of concrete fracture. To the best of our knowledge, such a simple and accurate prediction has only been accomplished by this new linear BEM.

Generally, this experimental observation reveals significant implications for fracture modeling of linear BEM. This model, supplemented with an average aggregate size $d_{av}$ of 5 mm and a 95% reliability band from the applied formula, was employed for validating fracture behavior across different specimen groups. Remarkably, the BEM reliably described the fracture loads of both small and large specimens, even in non-geometrically similar groupings, highlighting its adaptability as a predictive tool. This applicability has far-reaching consequences for the practical testing and modeling of concrete structures.

One key takeaway from Figure 1 is that fracture loads of large 3-P-B specimens or structures (un-notched or shallow-notched) can be accurately predicted using data from smaller notched specimens. For instance, fracture results from deep-notched small specimens (W=90 mm, $\frac{a_{0}}{W}=0.15\;\&\;0.3$) provided reliable predictions for the fracture behavior of large un-notched specimens (W=500 mm). Notably, even in large specimens with limited crack growth ($\frac{a_{0}}{W}=0.025$), the predictions based on the equivalent fracture area ($A_{e}$) aligned with experimental observations. This insight is substantial as it establishes a foundation for utilizing data from smaller and more manageable laboratory tests to predict the structural behavior of large-scale concrete components under real-world conditions.

**Figure 2:**
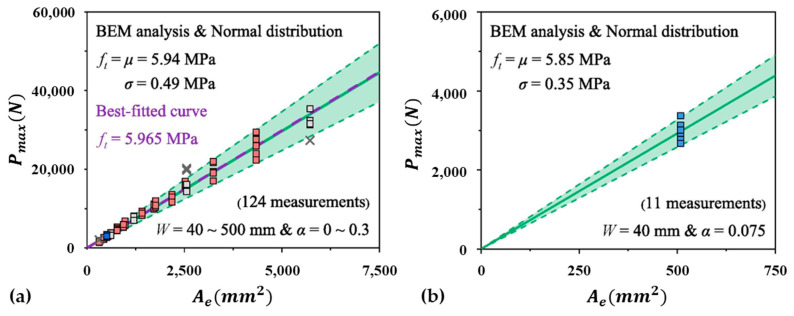
(**a**) *f_t_* from a total of 124 specimens with *W* from 40 to 500 mm and various notch lengths. (**b**) *f_t_* from 11 specimens of *W* = 40 mm.

As a comparison, the typical size effect experiments for reliable curve fitting require geometrically similar specimens, i.e., $\frac{a_{0}}{W}$ = constant, and the size range for W has to be as thick as probable. For instance, a total of 124 samples in two separate comprehensive concrete tests in the literature [14,15] chose the size span for W from 40 mm to 500 mm. The tensile strength $f_{t}$ calculated by BEM for a total of 124 samples is displayed in Figure 2a.

Three SELs are employed for curve fitting of geometrically comparable specimens: un-notched ($\frac{a_{0}}{W}=0$), shallow-notched ($\frac{a_{0}}{W}<0.1$) and deep-notched ($\frac{a_{0}}{W}>0.1$). Due to the stringent requirements of the SELs, only 11 single-group three-point bending tests were conducted for specimens with a size of W=40 mm and $\frac{a_{0}}{W}=0.075$. The estimation of tensile strength $f_{t}$ derived from this group of 11 specimens using BEM is illustrated in Figure 2b. The relative error between the two evaluations is below 2%. Once again, the results presented in Figure 2 [16] demonstrate that the quasi-brittle fracture behavior of concrete specimens with varying sizes (ranging from 40 to 500 mm) can be accurately predicted by the results from the smallest samples (40 mm).

In Figure 1, the estimated tensile strength $f_{t}$ is 5.9 MPa, with a relative error of less than 1% compared with the results shown in Figure 2. As consistent estimations of $f_{t}$ can be derived from different arrangements of the experimental outcomes presented in both figures, the strict requirement for geometric similarity in size effect experiments can be eliminated. This change will greatly simplify the process of conducting size effect experiments and modeling, as demonstrated in [16].

## 3. Materials and Methods

The above literature confirms that the strict size effect is not necessary for BEM analysis; therefore, in this study, a single set of geometric concrete specimens was designed. Commercially available concrete pavement units, specifically the Brighton Masonry $400\times 400\times 40\;{mm}^{3}$ Steel Mypave sourced from Bunnings (Perth, WA, Australia), were selected to ensure practicality and relevance to real-world applications. This selection ensures practicality and relevance to real-world applications; however, the actual thickness of these pavements measured approximately 34–39 mm due to their rough surface features.

The concrete pavements were cut into individual samples measuring $160\times 40\times \sim 38\;{mm}^{3}$ using a diamond blade concrete cutter, and surface notches were introduced with a hand-held cutter kit equipped with a 1 mm thick diamond blade. Notches of 1 mm and 6 mm were created in comparison with unnotched samples. Variations in notch size occurred due to the manual cutting process.

**Figure 3:**
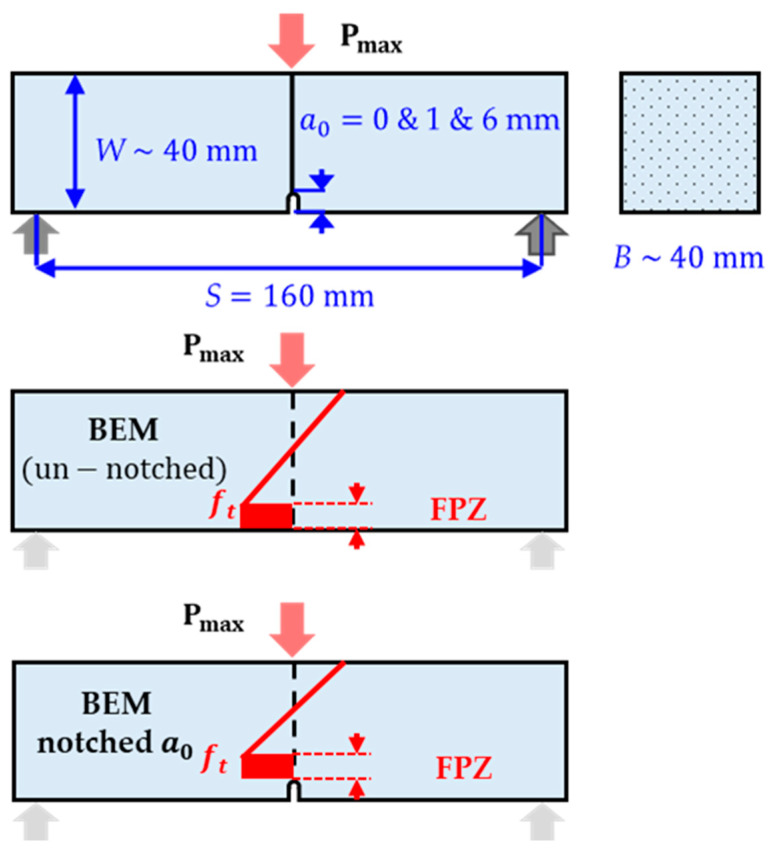
3-P-B specimen with known *FPZ* at the notch tip (Note: Arrows in this figure indicate the locations of the 3-P-B load cells and also reflect how stress is distributed on the specimens).

A series of three-point bending (3-P-B) tests as demonstrated in Figure 3 were conducted to investigate the tensile strength ($f_{t}$) of these commercial concrete plates with an unknown average particle size ($d_{av}$). The tests were performed using an Instron 5982 universal testing machine (Instron Corp., Norwood, CO, USA), with a testing rate of 1 mm/min and automatic recording of load and crosshead displacement every 0.1 s.

The specimen span (S) was maintained at 160 mm throughout the experiments, and all test samples presented variable width and thickness due to manual cuttings and instinct rough surfaces. For the 48 un-notched specimens analyzed, the thickness (B) ranged from 33.7mm to 40.9 mm, while the width (W) varied between 38.1 mm and 43.9 mm. This variability was primarily attributed to the cutting process but was reliably accounted for using a specific formulation, Equation (7). Additionally, shallow-notched specimens were prepared, consisting of 46 specimens with a notch depth ($a_{0}$) of 1 mm and exhibiting B values of 32.9 mm to 38.8 mm and W values ranging from 36.2 mm to 42.2 mm. For thorough comparison, 44 deep-notched specimens with $a_{0}=6\;mm$ were included in the test set, featuring a thickness range of 35.4 mm to 39.6 mm and widths spanning from 36.7 mm to 42.7 mm.

## 4. Results and Discussion

### 4.1. Development and Evolution of Size Effect Models: From SEL to BEM

The Size Effect Law (SEL) [10] has long been regarded as a foundational model for describing fracture behavior in brittle materials, particularly for deep-notched geometrically similar specimens. Its formulation predicts the nominal strength $\sigma_{N}$ using the maximum fracture load $P_{max}$ without considering the impacts of the fracture process zone (FPZ). The original SEL is expressed as follows:

(10) $$\sigma_{N}=\frac{B_{0}\times f_{t}}{\sqrt{1+\frac{W}{W_{0}}}}$$

where $B_{0}$, correlated to material strength, and $W_{0}$, a specific structural size, are fitting parameters obtained from experiments. However, a significant limitation of SEL arises because the tensile strength $f_{t}$ cannot be directly separated during curve fitting parameter $B_{0}$, restricting SEL’s predictive function to deep-notched specimens ($\frac{a_{0}}{W}>0.1$) with geometrically similar test samples. Furthermore, this SEL cannot account for the varying FPZ effects in shallow-notched or unnotched specimens, further restricting its applicability, as shown in [16].

The Boundary Effect Model (BEM) [16,19,28], introduced as an alternative formulation, expands on SEL to incorporate notched and unnotched specimens ($\frac{a_{0}}{W}>0$). Mathematically, the BEM nominal strength formula has a similar form [19]:

(11) $$\sigma_{N}=\frac{B_{0-BEM}\times f_{t}}{\sqrt{1+\frac{W}{W_{0-BEM}}}}\;$$

Similar to Equation (10), this BEM also contains two fitting parameters, $B_{0-BEM}$ and $W_{0-BEM}$. When Equations (10) and (11) are still managed for curve fitting, there is no progress from the SEL to the new proposed BEM. Although the modeling of size effects and the formulation of boundary effects stem from different concepts and assumptions, they ultimately yield the same results. The new linear BEM in Equation (1) with one parameter (material constant $f_{t}$) is simpler than in Equations (10) and (11). If the tensile strength $f_{t}$ is known, there is no need for curve fitting. As a result, the new linear BEM becomes a predictive model.

Additionally, it is important to note that Equation (2) within the context of SEL is restricted to deep-notched geometrically similar specimens where $\frac{a_{0}}{W}>0.1$ ($\frac{a_{0}}{W}=$ constant for a selected set of test samples). In contrast, the BEM specified in Equation (1) is applicable for any 3-P-B specimens, regardless of size or initial notch length, i.e., $\frac{a_{0}}{W}\geq 0$.

### 4.2. Aggregate Size Determination

It is noteworthy to recall again that one significant parameter under examination in fracture mechanics in the BEM is the fracture strength of concrete, which, in theory, is described as a material constant for a given concrete mix. Therefore, the fundamental assumption in this research is that tensile strength $f_{t}$ should remain constant, regardless of structural defects or notches introduced at the material interface. This study employed this constant theory and translated to the statistical equivalence of fracture strength in un-notched, shallow-notched (1 mm), and deep-notched (6 mm) specimens:

(12) $$f_{t}\left(a_{0}=0\right)=f_{t}\left(a_{0}=1\right)=f_{t}\left(a_{0}=6\right)$$

**Figure 4:**
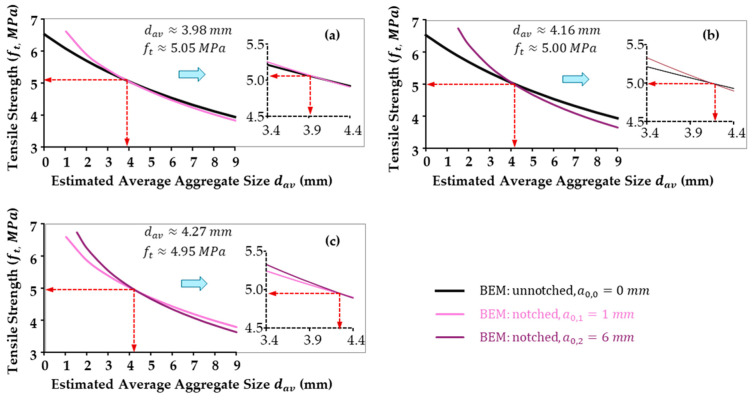
The statistical equivalence of fracture strength (material constant, $f_{t}$) in un-notched, shallow-notched, and deep-notched specimens computes $d_{av}$ values as 3.98 mm, 4.16 mm, and 4.27 mm—(**a**) $f_{t}\left(a_{0}=0\right)=f_{t}\left(a_{0}=1\right)$, (**b**) $f_{t}\left(a_{0}=0\right)=f_{t}\left(a_{0}=6\right)$, and (**c**) $\;f_{t}\left(a_{0}=1\right)=f_{t}\left(a_{0}=6\right)$. An average of 4.14 mm was adopted as the average aggregate size $d_{av}$ in this research.

Statistically, the fracture strength $f_{t}$ of each test sample was determined using Equation (7) with an assumed $d_{av}$ value. Then, three series of average tensile strengths $f_{t}$ for each group ($a_{0}=0,1,6)$ were obtained while altering the assumed average aggregate size. Mathematically equalized tensile strength and average aggregate size are obtained through Equation (12). As illustrated in Figure 4, both average fracture strength and average aggregate size (or effective aggregate size in this geometry analysis, $d_{av}$) were determined, where $d_{av}$ are 3.98 mm, 4.16 mm, and 4.27 mm, respectively. Thus, an average $d_{av}$ of 4.14 mm was adopted in this research for further fracture analysis and maximum failure load predictions.

**Figure 5:**
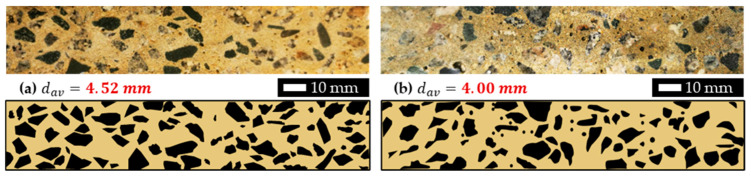
Concrete interface mapping where two samples were randomly selected and analyzed by Image J, which reveals average aggregate sizes are 4.52 mm and 4.00 mm.

To validate this aggregate size result, two concrete samples were randomly selected for detailed interface scanning, where surface sanding with fine mesh was performed, and then the concrete interface was mapped and analyzed using Image J software (https://imagej.net/ij/download.html, accessed on 23 July 2022). This process involved measuring the surface area and counting aggregates, as well as estimating aggregate area (or dark area, as demonstrated in Figure 5), to determine the average aggregate size. As the processing figure illustrated in Figure 5 and computed data summarized in Table 1, the aggregate sizes were measured to be 4.52 mm and 4.00 mm, with an average of 4.26 mm, which aligns closely with the values calculated through BEM. This strong correlation between experimental measurements and computational analysis emphasizes the reliability of the BEM formula.

It is important to repeat that the first step in the Boundary Effect Model (BEM) analysis for concrete structures is to examine the average aggregate size. As defined in Equation (7), for a given specimen geometry, the average aggregate size is the only independent variable used to compute tensile strength under a specific fracture failure load. In the context of BEM, this average aggregate size is referred to as the characteristic value, which is a crucial factor in determining structural failure. Structural failures in concrete typically arise from uneven stress distribution across the aggregates, making the accurate selection of this characteristic value essential. Additionally, when analyzing fiber-reinforced plastic (FRP) [24], the ply thickness is chosen because failures often occur along the plies.

Therefore, if the characteristic microstructure is improperly selected or evaluated, the effectiveness of the Boundary Effect Model (BEM) in predicting structural behavior may be compromised. This is particularly critical in the context of composite materials with multiple porosities [29] and multi-element nanocomposites [30], where even minor variations in pore diameter or grain size can lead to significant discrepancies in performance. In these cases, the scale of the selected characteristic microstructure is often too small to accurately represent the material’s behavior, which highlights the requirement for more sensitive and precise selection criteria. Careful evaluation of the characteristic value is essential to ensure that the BEM remains a reliable tool for predicting structural integrity in such complex composite materials.

### 4.3. Reliability Analysis for BEM

**Figure 6:**
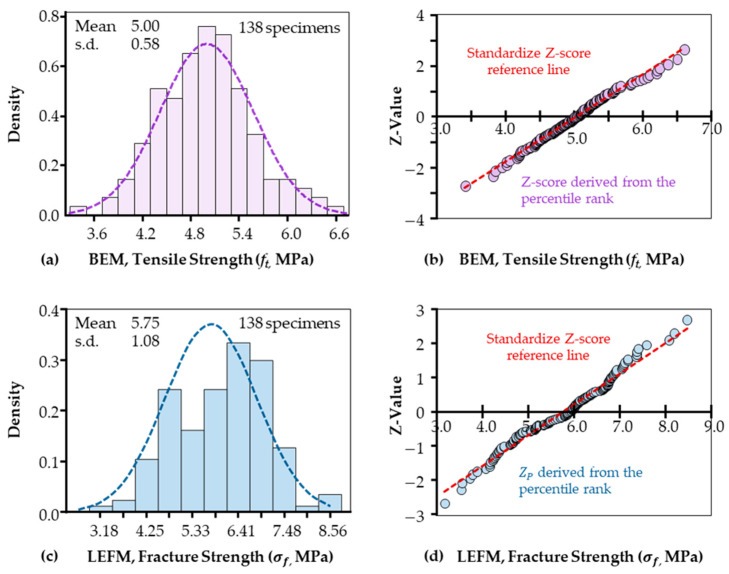
The tensile strength computed using the *BEM* model exhibits a central trend distribution and a linear Z-value relationship, confirming a normal distribution. Conversely, the fracture strength calculated via *LEFM* demonstrates non-normal distribution characteristics.

This study aimed to establish a statistically reliable methodology to predict the allowable load of industrial-scale concrete structures based on laboratory-obtained tensile strength data. Tensile strength values for 138 concrete samples were calculated using the BEM. In order to assess the normality nature and further verify the reliability of results calculated by BEM, a particular normality test was followed, and the resultant data distribution is demonstrated in Figure 6a. A standard bell curve was clearly presented, confirming the high reliability of this linear model. A range of 3.84 MPa to 6.16 MPa and 93.48% of original data was observed within ±2 s.d. range, with a mean and median tensile strength of 5.00 MPa and a standard deviation of 0.58 MPa. Its alignment with a normal distribution was further confirmed by the Z-value analysis in Figure 6b, which shows the data points form a linear fit.

Specifically, this Z-score demonstrates how many standard deviations the data point X ($f_{t}$ for BEM and $\sigma_{f}$ for LEFM) is from the mean value. The standard Z-score for a data point X is calculated using the following formula:

(13) $$Z=\frac{X-mean}{s.d.}$$

To evaluate if the dataset is normally distributed, a Z-score from the percentile rank ($Z_{p}$) is utilized to compare to the above-standardized Z values. $Z_{p}$ is the inverse CDF or quantile function of the normal distribution, denoting as $\Phi^{-1}\left(p\right)$ or $NORM.INV\left(p\right)$ in Excel, while the approximate cumulative probability (percentile, p) of the data point could be computed through (rank−0.5/n). Ideally, the Z-score test of original data points should align with the standard Z-score reference line if the test data are normally distributed. Additionally, statistical tests—including the Lilliefors test ($P_{value}=0.5$), Jarque-Bera test ($P_{value}=0.464$), and Anderson-Darling test ($P_{value}=0.567$)—were conducted in MATLAB (R2023b). All three tests confirmed that $f_{t}$ follows a normal distribution.

For comparison, the fracture strength of all unnotched and notched samples calculated using Linear Elastic Fracture Mechanics (LEFM) was assessed for normality via both visual inspection in Figure 6c and Z-value analysis in Figure 6d. The fracture strength $\sigma_{f}$ was calculated through Equation (8) (without considering notch influences on the notch tips). The dataset can fit a bell curve also; data density plots, however, present a right deviation. Statistically, it has a mean value of 5.75 MPa with a ±2 standard deviation range of 3.6 MPa to 7.9 MPa. Moreover, a non-(or less)-normality trend was observed using the Z-value plot as deviations from linearity in both end tails. Moreover, similar statistical normality checks were performed in MATLAB; however, one out of the three tests rejected normality. The test that rejected normality was the Anderson–Darling test ($P_{value}=0.0234$), which also indicated a lesser degree of normality in the LEFM-examined data. Furthermore, the wide data range observed raises concerns about the reliability of LEFM for predictive modeling, particularly in engineering structure survival assessments. Again, this unreliable result is well explained using BEM, as a particular FPZ is not considered in LEFM.

Instead, the analysis conducted using the BEM has demonstrated the reliability of this method by revealing a relatively narrow range of tensile strengths. This consistency aligns with the inherent uniformity of the material, as most samples performed within a consistent range near the mean of 5.00 MPa. It aligns with the manufacturing requirement that a six-sigma quality control methodology be employed in the concrete industry [31,32]. However, the presence of a rare lower-bound tensile strength value of 3.40 MPa warrants careful consideration for applications requiring extreme loading resistance. On the other hand, roughly around 50% of the samples exhibited tensile strength values exceeding 5 MPa, confirming the concrete’s suitability for moderate to high tensile load demands. With no values below 3 MPa, the baseline strength of the samples provides additional assurance of the material’s suitability for structural design under standard conditions.

Not surprisingly, the observed broader range of fracture strength ($\sigma_{f})$ calculated through Equation (8) is expected because—again—the fracture process zone (FPZ) effect is not accounted for stress concentration at the notch tip. Consequently, specimens containing notches—whether 1 mm or 6 mm—exhibit reduced fracture loads, leading to correspondingly lower fracture strength values. So, this classic equation (Equation (8)) is not suitable for fracture analysis of surface defects containing specimens.

### 4.4. Analysis of Test Results Based on BEM for P_max_ Prediction

According to the findings presented in Figure 1 and Figure 3, approximately 20 specimens are adequate to provide a reliable assessment of the tensile strength $f_{t}$. This estimation facilitates accurate forecasts of the maximum fracture load $P_{max}$ for various large three-point bending (3-P-B) specimens or constructions. By utilizing the linear BEM in conjunction with the statistical reliability limits indicated in Equation (9), a single, convenient size for small 3-P-B tests—regardless of whether they have an initial notch—has substantially streamlined the process of conducting size effect experiments and modeling.

**Figure 7:**
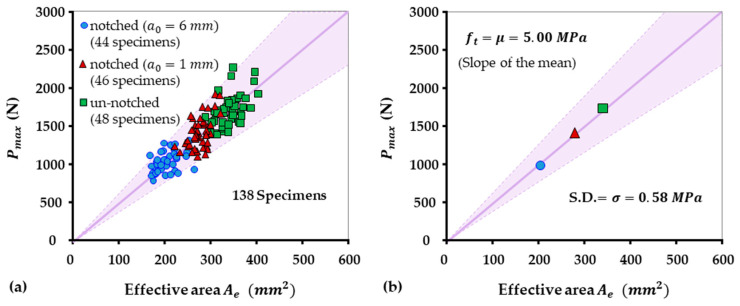
(**a**) Three groups of small 3-P-B test results for un-notched, shallow-notched (1 mm) and deep-notched (6 mm) specimens of constant size W (around 40 mm), as modelled by Equation (1) or (9). (**b**) The means from the three 3-P-B groups, showing the same tensile strength $f_{t}=5\;MPa$ (the slope of the mean line), is determined by the linear BEM.

In this research, in order to ensure the reliability of the findings and eliminate potential variances in the material properties that could affect tensile strength, all specimens were sourced from a single batch of concrete pavements. These pavements were manufactured on the same day, thus mitigating any variations in the composition, curing process, or other external factors. After all 3-P-B tests, equivalent area $A_{e}$ was calculated through Equation (2), and all data points were presented in Figure 7 with ±2σ reliability reference lines, as described in Equation (9). It is important to mention again that although the cutting process for these specimens exhibited inconsistency and slight size unevenness owing to an imprecisely controlled method, adherence to a consistent 160 mm span was maintained throughout the study. Interestingly, the randomly distributed size variations did not challenge the reliability of the tensile strength results. Given the calculated results of tensile strength, $f_{t}$ remained consistent and statistically similar—as evidenced in Figure 7a that most of the data points sitting within $f_{t}\pm 2\sigma$, reinforcing the fact that tensile strength is a material constant, essentially independent of minor size deviations.

According to the data dots distribution in Figure 7, it is clear that even tiny interfacial defects, such as a 1 mm notch, can significantly reduce the load-bearing capacity of the concrete specimen. This phenomenon can be effectively explained using the BEM, which provides a simple framework for analyzing the effects of stress concentrations and fracture processes at defect sites. Specifically, the presence of a fracture process zone, as described in Figure 3, which holds a distinct FPZ width (${FPZ}_{W}$) extending around the defect or notch tip, plays a critical role in weakening the mechanical load of the material. This ${FPZ}_{W}$ reduces the effective moment arm available to bear the applied load or stress, consequently decreasing the overall allowable stress the structure can sustain before structural failure happens. This allowable stress or maximum load is linear correlated to the tensile strength according to BEM, revealing that even small scale geometric interfacial cracks can lead to significant reductions in structural reliability.

Again, all the experimental results, along with ±2σ reliability band from Equation (9), are plotted in Figure 7a. Figure 7b displays the three mean values for un-notched, shallow-notched (1 mm), and deep-notched (6 mm) specimens. The minimal error across these three mean points reflects the large number of tests conducted. It also demonstrated that small specimens—regardless of whether they have notches (shallow or deep)—could effectively be used to determine the tensile strength $f_{t}$ from three-point bending (3-P-B) experiments via the BEM.

With the tensile strength $f_{t}$ established, Equation (9) serves as a predictive model, enabling the estimation of the maximum fracture load $P_{max}$ for any 3-P-B geometry and size. Additionally, a reliable predictive band can be expected to provide a reasonable bearing load range to enhance the reliability of the predicted values.

**Figure 8:**
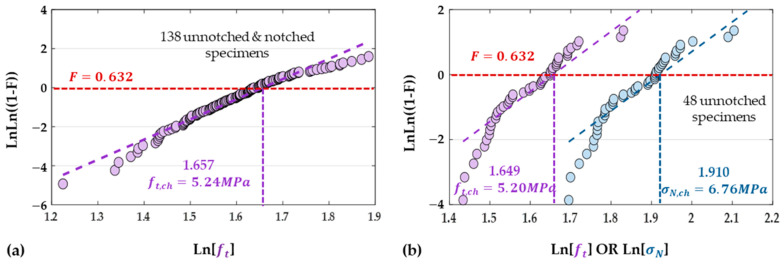
(**a**) Weibull distribution analysis of 138 un-notched and notched samples calculated by *BEM*; (**b**) Weibull distribution analysis of 48 unnotched samples using *BEM* (purple, tensile strength *f_t_*) and *LEFM* (blue, nominal strength *σ_N_*).

To further explore a more reliable technique to be adapted in this predictive model, a Weibull survival analysis containing the tensile strength value of all 138 samples was performed via MATLAB. In general, $F=1-exp\left\{-{\left(\frac{\sigma_{f}}{\sigma_{ch}}\right)}^{M}\right\}$ is applied for Weibull analysis, where a higher Weibull Modulus (M), typically above 5 MPa, indicates a well-controlled and consistent process. In this study, the fitted modulus of 5.26 MPa confirms the consistent concrete manufacturing process again. As demonstrated in Figure 8a, the $LnLn\left(1-F\right)\;\mathrm{vs}.\;Ln(f_{t})$ plot demonstrates a strong Weibull fit, with a characteristic strength of 5.24 MPa (F=0.632). Typically, if this characteristic strength with a 63.2% failure probability is utilized in engineering design, the maximum fracture load can be predicted as $5.24\times A_{e}$ for a given geometrical engineering beam with the same concrete mix. Additionally, a Weibull analysis of 48 unnotched samples was performed, as shown in Figure 8b, yielding a characteristic strength of 5.20 MPa. Compared with the result (5.24 MPa) in Figure 8a, only a 0.76% error was observed, further validating the effectiveness of the BEM for all specimens, irrespective of the presence of surface defects.

As discussed, LEFM is not ideal for specimens containing surface flaws, thus for comparison, only the nominal strength $\sigma_{N}$ of above the 48 unnotched samples was calculated by Equation (8) and then followed by a similar Weibull analysis, as demonstrated by blue trends in Figure 8b. According to Equations (7) and (8), nominal strength $\sigma_{N}$ and tensile strength $f_{t}$ correlates to the following:

(14) $$\frac{\sigma_{N}}{f_{t}}=\frac{\left(1+\frac{{3d}_{av}}{W}\right)}{1}\to \sigma_{N}=(1+\frac{3d_{av}}{W})\times f_{t}$$

With an average aggregate size $d_{av}$ of 4.14 mm and specimen thickness W of roughly 40 mm, a correlation factor of 23.7% was derived. This relationship is further validated by a 23.05% difference observed between the characteristic nominal strength and characteristic tensile strength, as illustrated in Figure 8b. That is, tensile strength can be effectively computed using Equation (14) if nominal strength is specified in the datasheet. Therefore, a reliable maximum load prediction could be followed by BEM. This approach allows reliable predictions of fracture loads even without any tests.

In general, it is well confirmed the estimated BEM, as referenced in Equation (1), is perfectly established to enable the prediction of maximum bearing load $P_{max}$ on large geometric concrete beams using tensile strength data obtained from small samples. Significantly, this BEM has been further studied for the evaluation of crack initiation load $P_{i}$ in a recent study [22] by ideally removing the ${FPZ}_{L}$ impact within Equation (2) $({FPZ}_{L}=0).$ This advancement allows for a comprehensive prediction of the entire lifecycle of a given concrete beam from crack initiation to ultimate failure at maximum load.

### 4.5. Notch Width Influence on K_IC_

Usually, the initial crack presented on concrete samples does not feature a sharp crack tip but instead has a width of 1–4 mm. Yet, if the notch width $n_{w}$ is relatively tight compared to the fracture process zone width ${FPZ}_{W}$ (approximately three times the average aggregate size or ${3d}_{av}$), the influence of the notch width can be assessed using the following relationship [18,25]:

(15) $$K_{IC}\approx K_{IC-NM}\times \sqrt{\frac{{3d}_{av}}{{3d}_{av}+n_{W}}}$$

In this research, with a notch width $n_{w}$ of 1 mm and $d_{av}=4.14\;mm$, Equation (15) indicates that the toughness relation is 96.20%. Consequently, the impact of notch width can be considered negligible.

The simple estimate of the notch effect in Equation (15) is feasible primarily because the fracture process zone width ${FPZ}_{W}$ is developed in Equations (1) and (2). Most of the fracture analysis focus on the influence of ${FPZ}_{L}$; thus, the notch width effect described in Equation (15) is distinctive to BEM, which accounts for both ${FPZ}_{L}$ and ${FPZ}_{W}$.

## 5. Conclusions

It has been four decades since the original Size Effect Law (SEL) [9,10] was proposed for the quasi-brittle fracture of concrete and over 20 years since the introduction of the Boundary Effect Model (BEM) to study interactions between the fracture process zone (FPZ) and specimen boundaries [19]. After so many years of intensive studies, substantial progress in size effect modeling and experimental methods should be expected.

The recent work on the interchangeability of SEL and BEM [16] has demonstrated that the three SELs with multiple fitting parameters can be simplified to a single linear function, as expressed in Equation (1). This new linear BEM formulation is fully characterized by two key parameters: the aggregate size $d_{av}$ and the tensile strength $f_{t}$ which serve as the criteria for FPZ formation in both notched and un-notched specimens.

Through the new linear BEM adopted in this study, it is proven that the transition from the Size Effect Law (SEL) to the Boundary Effect Model (BEM) has significantly the simplified modeling of quasi-brittle fracture of concrete. Different from curve-fitting via three SELs, the linear BEM is a predictive model with the well-defined tensile strength $f_{t}$ (criterion for FPZ formation) and the average aggregate size $d_{av}$. Statistical analysis of 138 notched and un-notched concrete samples yielded a tensile strength with a mean of 5.00 MPa and a standard deviation of 0.58 MPa. We confirmed the reliability of the BEM through a normal distribution analysis and a Weibull analysis with a characteristic strength of 5.24 MPa. Additionally, the predictions for maximum fracture loads are effectively captured by the linear BEM; this explains its applicability across various specimen geometries regardless of size effect.

Furthermore, with a notch width $n_{w}$ of 1 mm and $d_{av}=4.14\;mm$, the calculated toughness relation for a 1 mm notch width was found to be approximately 96.20% according to Equation (15). This supports the notion that notch width in this study has a minimal impact on fracture toughness. The accuracy, simplicity and effectiveness of the linear BEM is desirable for practical engineering applications and research studies at universities.

The simplicity of Equation (9), together with its statistical functionality, can potentially be utilized as a predictive safety design tool for engineering structures. In other words, the maximum load of a structure can be confidently predicted with a reliable range, as discussed in this study. If needed, the initial micro-cracking load at which crack initiation occurs can also be determined using BEM, as discussed in [22]. This predictive linear BEM model distinguishes itself from the numerous models previously proposed for concrete fracture [9,10,11,12,13,15,33,34,35,36,37,38].

The new “Hall-Petch” relation for brittle solids and composites, as given in Equation (6), is critical for the derivation of the linear BEM shown in Equations (1) and (7), the classic Hall–Petch relation for metals, commonly explained in textbooks, is limited to the grain size range from 20 nm to 200 μm, the new “Hall-Petch” relation for brittle solids and composites is valid for microstructures from the atomic scale (<1 nm) to around 200 mm for dam concrete. Furthermore, the new “Hall-Petch” relation covers various brittle solids and composites, as summarized in [18]. Therefore, it is expected that the new “Hall-Petch” relation for brittle solids will play a more significant role in fracture modeling and explanation of the intrinsic linkage between strength and toughness.

## Figures and Tables

**Table 1:** Concrete sample surface mapping used to determine average aggregate size.

(a) Sample 1	
Aggregate Count	84
% Area	35.7
Original Sample Area	160 mm×30 mm
Average Aggregate Size	4.52 mm
(b) Sample 2	
Aggregate Count	88
% Area	29.29
Original Sample Area	160 mm×30 mm
Average Aggregate Size	4.00 mm
Analysed by Image J	

## Data Availability

The raw data supporting the conclusions of this article will be made available by the authors on request.

## Abbreviations

The following abbreviations are used in this manuscript:

*BEM*	Boundary Effect Model
*SEL*	Size Effect Law
*FPZ*	Fracture Process Zone
*LEFM*	Linear Elastic Fracture Mechanics
